# Morphological Spectrum and Survival Analysis of Diffuse Midline Glioma With H3K27M Mutation

**DOI:** 10.7759/cureus.17267

**Published:** 2021-08-17

**Authors:** Usman Hassan, Maliha Latif, Irfan Yousaf, Saad Bin Anees, Sajid Mushtaq, Noreen Akhtar, Asif Loya

**Affiliations:** 1 Pathology, Shaukat Khanum Memorial Cancer Hospital and Research Centre, Lahore, PAK; 2 Histopathology, Shaukat Khanum Memorial Cancer Hospital and Research Centre, Lahore, PAK; 3 Surgery, Shaukat Khanum Memorial Cancer Hospital and Research Centre, Lahore, PAK

**Keywords:** diffuse midline glioma, h3-k27m mutation, histological, immuno-histochemical, morphological spectrum

## Abstract

Background

Diffuse midline gliomas with the H3K27M mutation are now recognized as separate entities due to their unique molecular signature, clinical features, and adverse outcome.

Objective

To determine the morphological spectrum and survival rate of diffuse midline gliomas with H3K27M mutation.

Material and methods

This retrospective study was conducted between January 2015 and January 2021 at Shaukat Khanum Memorial Cancer Hospital and Research Centre. Medical records of 28 cases of H3K27M-mutated midline gliomas were retrieved. Case slides were reviewed and the pertinent histological spectrum was evaluated.

Results

The mean age of patients was 24.36 ± 14.06 years. There were 21 (75%) males and 7 (25%) females. Biopsy was performed in 22 (78.6%), total resection in 1 (3.6%) while subtotal resection was done in 5 (17.9%) cases. Histologically, a spectrum of morphologies was noted with pilocytic astrocytoma (WHO grade 1) at one end and glioblastoma (WHO grade IV) at the other end. Immunohistochemically, all 28 cases were positive for Histone 3 immunohistochemistry. ATRX was performed in 7 (25.0%) cases with loss of ATRX expression in 3 (10.7%) and retained expression in 4 (14.3%) cases. Ki67 was <5% in 6 (21.4%), 5-10% in 1 (3.6%), 11-15% in 1 (3.6%), 16-20% in 3 (10.7%), 21-25% in 4 (14.3%), and 26-30% in 2 (7.1%) cases. The mean survival was 8.00 ± 9.39 months. Out of 28 patients, 15 (62.5%) patients died of disease.

Conclusion

Diffuse midline gliomas with H3K27M mutation is an aggressive entity with a broad morphological spectrum.

## Introduction

Gliomas are the most frequent primary tumors of the CNS and encompass two principal subgroups: diffuse gliomas and gliomas showing a more circumscribed growth pattern (non-diffuse gliomas) [1]. Recent molecular studies have revolutionized our understanding of high-grade gliomas (HGG). The recent WHO categorization of brain tumors puts forward the concept of integrated diagnosis [2]. Modern diagnostic amendments have received greater attention in the last few years and now the histological diagnosis of brain tumors is being made in combination with molecular testing. This has resulted in the emergence of novel entities like "diffuse midline gliomas with H3 K27M mutation" [3].

Due to their unique molecular signature and clinical features, diffuse midline gliomas with H3K27M mutation are now recognized as a separate entity in the 2016 WHO Classification of Tumors of the CNS. These tumors are usually found in the midline locations, such as the brainstem, thalamus, cerebellum, and spinal cord. H3K27M mutation was initially described in the pediatric age group, but afterward, it was also observed in the adult population [4].

The presence of H3K27M mutation in diffuse intrinsic pontine glioma (DIPG) confers a worse prognosis [3,5]. Extrapolating from the clinicopathologic features of DIPGs and the poor prognosis, H3K27M-mutated glioma shows a worse prognosis regardless of the lesion's histological grade [6]. In the revised 2016 WHO classification, diffuse midline glioma with H3K27M mutation is recognized as a distinct entity that corresponds to grade IV, even when brisk mitotic activity, microvascular proliferation, and necrosis are not observed [7].

In some patients with H3K27 mutant tumors with alternate morphologies, more prolonged survival has been reported, making grading difficult for a neuropathologist [8]. The time before an occurrence occurs is the outcome variable of interest in survival analysis, which is a set of statistical procedures for data analysis. It is the analysis of the period between the start of observation and the occurrence of a subsequent event. Initial research, where the occurrence of concern was death, gave rise to the term "survival study” [8].

The aim of our study is to analyze the demographic and histological spectrum and clinical outcome of H3K27-mutated malignancies across both the pediatric and adult age groups. It would be the first of its kind in our south Asian population.

## Materials and methods

This retrospective study was conducted at Shaukat Khanum Memorial Cancer Hospital and Research Centre, Lahore, Pakistan. Approval from Institutional Review Board (IRB) was obtained prior to commencement of the study (IRB# EX-30-12-19-01). Cases reported as diffuse midline gliomas with the H3K27M mutation between January 2015 and January 2020 were included and followed up with the treatment at our center. Medical records of 28 cases with H327M-mutated midline gliomas, diagnosed on histological and immunohistochemical examination, were retrieved and included in the study. Patients with ages less than 16 years were included in the pediatric age group.

Non-midline gliomas, gliomas with negative or equivocal H3K27 mutation, and cases with incomplete clinical and radiological findings were excluded from the study group. All the relevant information like age, gender, site of the tumor, functional class of tumor, treatment, survival, complications, morphological and histopathological spectrum were obtained from the medical record. Case slides were reviewed and pertinent histological findings were re-confirmed.

Histone H3K27M immunohistochemical polyclonal antibody was used with the heat induced epitope retrieval (HIER) antigen retrieval method. Antigen retrieval time was 20 minutes and incubation time was 15 minutes. A bond polymer detection kit was used, and the expected positive staining pattern was nuclear.

Data was collected and analyzed using SPSS version 23. Quantitative variables were presented as mean and SD, while the categorical variables were presented as frequency and percentage.

## Results

The overall mean age of patients in our study was 24.36 ± 14.06 years. The mean age of pediatric patients was 12.33 ± 3.08, and the mean age of adult patients was 30.05 ± 13.63. There were 21 (75%) males and 7 (25%) females. There were 9 pediatric age group patients, of which 7 (77.8%) were male and 2 (22.8%) were female.

The pre-treatment functional class results showed that in pediatric patients two (25.0%) had functional class 1, one (12.5%) had functional class 2, two (25.9%) had functional class 3, and three (37.5%) had functional class 4. Similarly, in adult patients, three (18.8%) had functional class 1, five (31.3%) had functional class 2, five (31.3%) had functional class 3, and three (18.8%) had functional class 4.

Three (10.7%) patients had tumors in the brain stem, three (10.7%) in the intraventricular area, 12 (42.9%) in the thalamus, two (7.1%) in the spinal cord, and eight (28.6%) in other sites that were not specified. Biopsy was done in 22 (78.6%) cases, gross total resection in one (3.6%) case, while subtotal resection was done in five (17.9%) cases.

In pediatric patients, only surgery was performed in three (33.3%) patients, surgery with external beam radiotherapy (XRT) in three (33.3%), surgery with chemotherapy in one (11.1%), and surgery along with both XRT and chemotherapy in two (22.2%) cases. Similarly, in adult patients, treatment by only surgery was done in 10 (52.6 %) patients, surgery with XRT in seven (36.8 %), surgery with chemotherapy in one (5.3%), and surgery with both XRT and chemotherapy in one (5.3 %) case (Table 1).

**Table 1:** Demographic and clinical variables. XRT: External beam radiotherapy.

Study variables		Pediatric	Adults	Total
Age		12.33 ± 3.08	30.05 ± 13.63	24.36 ± 14.06
Survival in months		4.13 ± 3.98	10.00 ± 10.75	8.04 ± 9.39
Radiological progression in months		3.00 ± 3.51	4.94 ± 9.60	4.29 ± 8.05
Gender	Male	7 (77.8%)	14 (73.7%)	21 (75.0%)
	Female	2 (22.2%)	5 (26.3%)	7 (25.0%)
Functional class	1	2 (25.0%)	3 (18.8%)	5 (20.8%)
	2	1 (12.5%)	5 (31.3%)	6 (25.0%)
	3	2 (25.0%)	5 (31.3%)	7 (29.2%)
	4	3 (37.5%	3 (18.8%)	6 (25.0%)
Site of tumor	Thalamus	2 (22.2%)	10 (52.6%)	12 (42.9%)
	Brainstem	3 (33.3%)	0 (0.0%)	3 (10.7%)
	Intraventricular	1 (11.1%)	2 (10.5%)	3 (10.7%)
	Spinal cord	1 (11.1%)	1 (5.3%)	2 (7.1%)
	Others	2 (22.2%)	6 (31.6%)	8 (28.6%)
Surgery	Biopsy	7 (77.8%)	15 (78.9%)	22 (78.6%)
	Subtotal	2 (22.2%)	3 (15.8%)	5 (17.9%)
	Gross total	0 (0.0%)	1 (5.3%)	1 (3.6%)
Treatment	Surgery only	3 (33.3%)	10 (52.6%)	13 (46.4%)
	Surgery + XRT	3 (33.3%)	7 (36.8%)	10 (35.7%)
	Surgery + chemotherapy	1 (11.1%)	1 (5.3%)	2 (7.1%)
	Surgery + XRT + chemotherapy	2 (22.2%)	1 (5.3%)	3 (10.7%)
Complications	Neurological deficits	5 (62.5%)	9 (56.3%)	14 (58.3%)
	Seizures	2 (25.0%)	5 (31.3%)	7 (29.2%)
	Hydrocephalous	1 (12.5%)	2 (12.5%)	3 (12.5%)
Outcome	Dead	6 (75.0%)	9 (56.3%)	15 (62.5%)
	Alive	2 (25.0%)	7 (43.8%)	9 (37.5%)

The mean duration of survival was 8.00 ± 9.39 months (4.13 ± 3.98 in pediatric patients and 10.00 ± 10.75 in adult patients).

**Figure 1:**
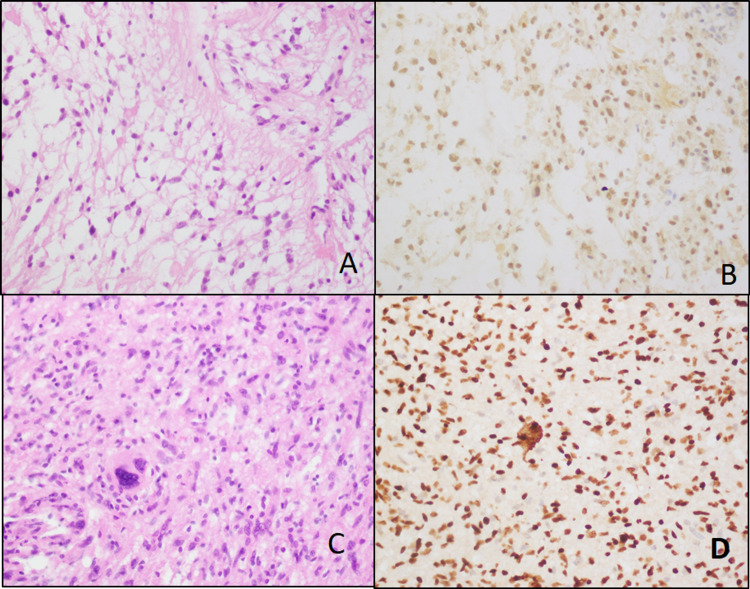
Grade 1 tumors. A & B: Pilocytic astrocytoma-like histology and histone 3 IHC stain (40X). C & D: Pleomorphic xanthoastrocytoma-like histology and histone 3 IHC stain (40X). IHC: Immunohistochemistry.

**Figure 2:**
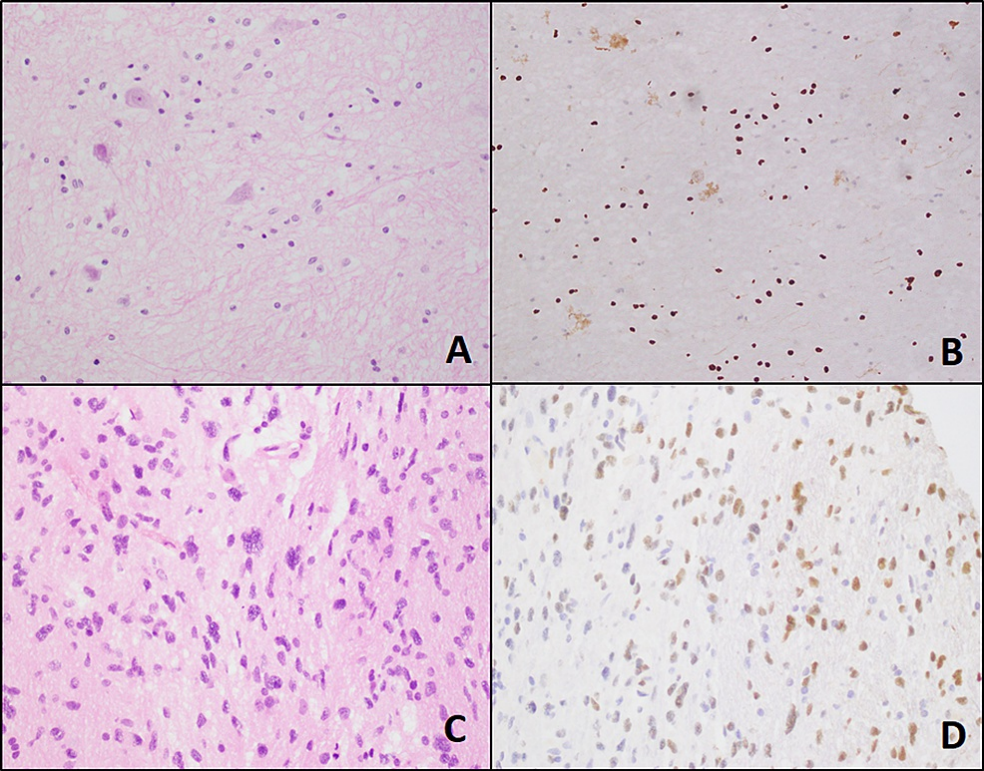
Grade 2 tumors. A & B: Ganglioglioma-like histology and histone 3 IHC stain (40X). C &D: Astrocytoma-like histology and histone 3 IHC stain (40X). IHC: Immunohistochemistry.

**Figure 3:**
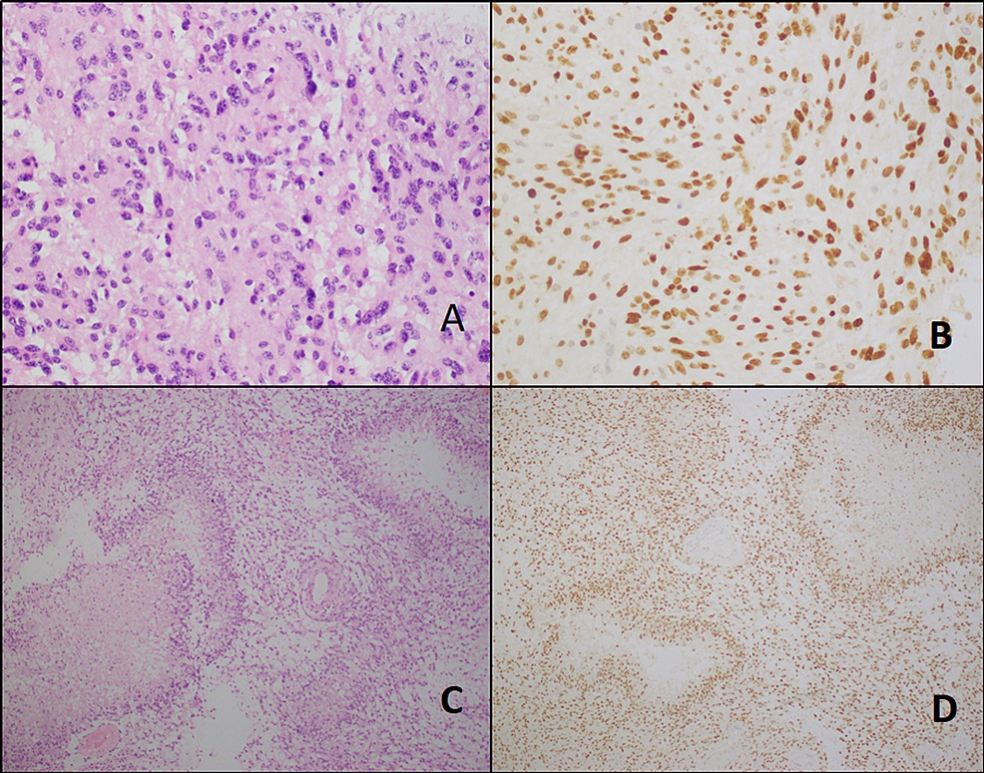
Grade 3 and 4 tumors. A & B: Anaplastic astrocytoma (grade 3)-like histology and histone 3 IHC stain (40X). C & D: Glioblastoma (grade 4)-like histology and histone 3 IHC stain (20X). IHC: Immunohistochemistry.

Histology slides were reviewed and morphological results showed that pleomorphic xanthoastrocytoma in two (7.1%) and pilocytic astrocytoma, WHO grade 1 morphology was noted in one (3.6%) case (Figure 1), ganglioglioma, WHO Grade-II morphology in one (3.6%) and diffuse astrocytoma, Grade-II morphology in six (21.4%) (Figure 2), anaplastic astrocytoma, WHO Grade-III morphology was noted in three (10.7%) cases, and glioblastoma WHO Grade-IV morphology in fifteen (53.6%) (Figure 3).

Of the 28 cases, three (12.5%) developed hydrocephalus, 14 (58.3%) had neurological deficits, and seven (29.2%) had seizures. Fifteen (62.5%) of 28 patients died of disease. Post-treatment change in functional class was -2 in four (16.7%) cases, -1 in six (25.0%) cases, 0 in 13 (54.2%), and 4 in one (4.2%) case.

Immunohistochemically, histone 3 was nuclear positive in all 28 (100%) cases. ATRX was performed in seven (25%) cases of which loss of expression was noted in three (10.7%) and retained expression in four (14.3%) cases. Ki67 expression was performed in 17 (60.7%) cases of which <5% proliferation index was noted in six (21.4%), 5-10% in one (3.6%), 11-15% in one (3.6%), 16-20% in three (10.7%), 21-25% in four (14.3%), and 26-30% in two (7.1%) cases (Table 2).

**Table 2:** Histopathological features of tumors.

Change in functional class post treatment	-2	2 (25.0%)	2 (12.5%)	4 (16.7%)
	-1	1 (12.5%)	5 (31.3%)	6 (25.0%)
	0	4 (50.0%)	9 (56.3%)	13 (54.2%)
	4	1 (12.5%)	0 (0.0%)	1 (4.2%)
Histopathology	Anaplastic astrocytoma, WHO grade-III	1 (11.1%)	2 (10.5%)	3 (10.7%)
	Diffuse astrocytoma, WHO grade-II	1 (11.1%)	5 (26.3%)	6 (21.4%)
	Glioblastoma, WHO grade-IV	6 (66.7%)	9 (47.4%)	15 (53.6%)
	Ganglioglioma, WHO grade-II	1 (11.1%)	0 (0.0%)	1 (3.6%)
	Pleomorphic xanthoastrocytoma, WHO grade-II	0 (0.0%)	2 (10.5%)	2 (7.1%)
	Pilocytic Astrocytoma, WHO grade 1	0 (0.0%)	1 (5.3%)	1 (3.6%)
ATRX	Loss of expression	0 (0.0%)	3 (15.8%)	3 (10.7%)
	Retained	1 (11.1%)	3 (15.8%)	4 (14.3%)
	Not performed	8 (88.9%)	13 (68.4%)	21 (75.0%)
Ki67	<5 %	0 (0.0%)	6 (31.6%)	6 (21.4%)
	5-10%	0 (0.0%)	1 (5.3%)	1 (3.6%)
	11-15%	0 (0.0%)	1 (5.3%)	1 (3.6%)
	16-20%	2 (22.2%)	1 (5.3%)	3 (10.7%)
	21-25%	1 (11.1%)	3 (15.8%)	4 (14.3%)
	26-30%	1 (11.1%)	1 (5.3%)	2 (7.1%)
	Not performed	5 (55.6%)	6 (31.6%)	11 (39.3%)

## Discussion

H3K27M mutation was initially described in pediatric DIPGs but now has been recognized in adult midline diffuse gliomas and other midline tumors with varying morphologies like gangliogliomas, anaplastic gangliogliomas, pilocytic astrocytomas, and posterior fossa ependymomas [8]. The presence of H3K27M mutation in DIPGs was recognized to portray an adverse prognosis regardless of the histological grade of the lesion and as a result, were directly assigned a grade IV in the 2016 WHO Classification [6,9].

H3K27M mutations are common in midline gliomas of adults; thus, this molecular subtype should be considered in adults of all ages and tumor grades. H3K27M mutations are associated with improved survival in adults, which is quite the opposite from pediatric patients where H3K27M mutations confer a worse prognosis [10].

In our study, histone H3K27M mutation was frequently reported in most diffuse midline gliomas. However, the relationship between clinical outcomes of H3K27M mutation and gliomas from different anatomical locations is still not fully understood [11]. H3K27M mutation in histone 3 has been described to identify high-grade midline gliomas associated with a particularly unfavorable prognosis [12].

In our study, the mean duration of survival was 8.00 ± 9.39 months. Out of 28, 15 (62.5%) cases died. The mean duration of survival in pediatric patients was 4.13 ± 3.98 and the mean duration of survival in adult patients was 10.00 ± 10.75. It was reported in a previous study by Kleinschmidt-DeMasters BK and Mulcahy Levy JM that the mean survival was 9.3 months for adults and 8.9 months for pediatric patients [8].

In our study, ATRX had a loss of expression in three (10.7%) and retained in four (14.3%). Ki67 expression was <5% in six (21.4%), 5-10% in one (3.6%) case, 11-15% in one (3.6%) case, 16-20% in three (10.7%) cases, 21-25% in four (14.3%) case, 26-30% in two (7.1%) case and not performed in 11 (39.3%) cases.

Another study by Aihara K, et al. of adult gliomas limited to the thalamus revealed an ATRX mutation in 29% of the tumors while in our study it was 25% and was not just limited to thalamus but also included brainstem, intraventricular, spinal cord, and other unspecified locations [13]. ATRX loss has been reported almost exclusively in isocitrate dehydrogenase (IDH)-mutant tumors in adults [14].

Given the novelty and rarity of the H3K27M mutation in adult brain tumors, the incidence and clinical behavior of these tumors in adults are still relatively unknown. The present data on H3K27M-mutated tumors in adults suggests that this mutation may be more prevalent in tumors of the spinal cord or thalamus in adults in contrast to pediatric patients [4,7,15,11]. Two series on midline gliomas showed an incidence of 50-60% H3K27M mutations in midline gliomas in adults located in the brainstem or thalamus [11,16].

The total proportion of H3K27M mutation in adult midline gliomas remains unclear. Besides, there is a lack of clarity regarding the clinical behavior of these tumors in adults and how their behavior differs from H3 wild-type gliomas in the same location. Currently, adult cases of diffuse midline gliomas are generally treated as their pediatric counterparts and merit a WHO grading of IV, irrespective of the actual grade found in microscopy, though not all series support this approach [16,17].

## Conclusions

Gliomas are prevalent both in pediatric and adult populations. Regardless of morphological features and grade of tumor, these tumors behave aggressively and have a bad prognosis, especially in children.

Diffuse midline gliomas with the histone H3K27M mutation may have a broad range of morphological characteristics. IHC is imperative for the identification of histone H3K27M mutation in all diffuse midline gliomas.
